# Syrbactin-class dual constitutive- and immuno-proteasome inhibitor TIR-199 impedes myeloma-mediated bone degeneration *in vivo*

**DOI:** 10.1042/BSR20212721

**Published:** 2022-02-11

**Authors:** Vasudha Tandon, Ruturajsinh M. Vala, Albert Chen, Robert L. Sah, Hitendra M. Patel, Michael C. Pirrung, Sourav Banerjee

**Affiliations:** 1 Department of Cellular Medicine, School of Medicine, University of Dundee, Dundee DD1 9SY, U.K.; 2 Department of Chemistry, Sardar Patel University, Vallabh Vidyanagar 388120, Gujarat, India; 3 Department of Bioengineering, University of California, San Diego, La Jolla, CA 92093, U.S.A.; 4 Department of Chemistry, University of California, Riverside, CA 92521, U.S.A.; 5 Department of Pharmaceutical Sciences, University of California, Irvine, CA 92697, U.S.A.

**Keywords:** breast cancers, cancer, drug resistance, enzyme activity, multiple myeloma, proteasome inhibitor

## Abstract

Proteasome-addicted neoplastic malignancies present a considerable refractory and relapsed phenotype with patients exhibiting drug resistance and high mortality rates. To counter this global problem, novel proteasome-based therapies are being developed. In the current study, we extensively characterize TIR-199, a syrbactin-class proteasome inhibitor derived from a plant virulence factor of bacterium *Pseudomonas syringae pv syringae*. We report that TIR-199 is a potent constitutive and immunoproteasome inhibitor, capable of inducing cell death in multiple myeloma, triple-negative breast cancer, (TNBC) and non-small cell lung cancer lines. TIR-199 also effectively inhibits the proteasome in primary myeloma cells of patients, and bypasses the PSMB5 A49T+A50V bortezomib-resistant mutant. TIR-199 treatment leads to accumulation of canonical proteasome substrates in cells, it is specific, and does not inhibit 50 other enzymes tested *in vitro*. The drug exhibits synergistic cytotoxicity in combination with proteasome-activating kinase DYRK2 inhibitor LDN192960. Furthermore, low-doses of TIR-199 exhibits *in vivo* activity by delaying myeloma-mediated bone degeneration in a mouse xenograft model. Together, our data indicates that proteasome inhibitor TIR-199 could indeed be a promising next-generation drug within the repertoire of proteasome-based therapeutics.

## Introduction

The proteolytic subunits β1/PSMB1/caspase-like, β2/PSMB2/tryptic-like, and β5/PSMB5/chymotryptic-like, represent the catalytic core of the 20S constitutive proteasome [1]. The 20S catalytic core associates with 19S regulatory complex to form the constitutive 26S proteasome [2]. In vertebrates, specific catalytically active β subunits, i.e., β1i/LMP2/PSMB9, β2i/MECL1/PSMB10, and β5i/LMP7/PSMB8, are incorporated into the 20S core proteasome upon interferon IFNγ-mediated induction to form the immunoproteasome [3]. The constitutive 26S proteasome degrades majority of cellular proteins in eukaryotes [1], while the immunoproteasome generates peptides from antigens to present to cytotoxic T cells [4]. Over the years, the proteasome has been pharmacologically targeted for the treatment of multiple myeloma [1] and mantle-cell lymphoma [5]. FDA-approved proteasome inhibitor bortezomib directly inhibits the 20S core of the proteasome and have significantly improved life expectancy of multiple myeloma patients [6], although with reported side effects and eventual drug resistance [7]. Bortezomib exhibits highest binding affinity to the PSMB5 subunit of the 20S proteasome; however, unfortunately, patients often develop bortezomib resistance due to various factors [8,9]. To circumvent the issues of bortezomib resistance and toxicity, immunoproteasome inhibitors like ONX-0914 or dual 26S and immuno-proteasome inhibitors are being developed preclinically albeit they do exhibit toxicities in patients [10,11]. ONX-0914 preferentially binds to the PSMB8 subunit of the immunoproteasome and bypasses bortezomib resistance in myeloma cell lines [12]. However, both these epoxyketone class molecules exhibit similar binding mechanism to carfilzomib, which eventually might lead to resistance [13]. Furthermore, various recent studies including our own have shown that multiple kinases can phosphorylate and regulate the activity of the constitutive proteasome [14–17]. Interestingly, targeting one such proteasome-regulating kinase, DYRK2, *in vivo* dramatically reduced tumor burden in multiple myeloma and triple-negative breast cancer (TNBC) mouse models [18–21]. In fact DYRK2 inhibitor LDN192960 significantly reduces proteasome activity and impedes systemic bone degeneration in immunocompetent mouse myeloma models [20] and induces synergistic cytotoxicity in cancer cells in combination with proteasome inhibitors [18,20]. Pharmacological targeting of such upstream kinases has indeed opened up new possibilities in proteasome-based therapeutics [18,20]. Therapeutic targeting of the 26S proteasome could also be beneficial for solid tumors like TNBC patients, [22,23] and in some cases for KRAS G12D lung cancer patients [24]. On a similar note, immunoproteasome inhibitors are also being developed to target various autoimmune, cancer, and inflammatory diseases [25]. Interestingly, various groups have reported that a simultaneous constitutive and immunoproteasome inhibition could in fact have an additive/synergistic effect in alleviating refractory/relapsed multiple myeloma progression [12,26]. Thus, a dual constitutive and immuno-proteasome inhibitor could be a major therapeutic blockbuster in the future.

In 2008, a plant virulence factor of bacterium *Pseudomonas* was found to directly bind and inhibit the proteasome [27]. The virulence factor derivatives called syrbactins are a family of bacterial, macrocyclic, non-ribosomal peptide natural products which react covalently with the catalytic Thr^1^ residue of proteasome peptidase subunits [27–29]. Upon further structure–activity relationship studies, syrbactin analog TIR-199 was developed and found to inhibit proteasome activity [30] and induce cell death in myeloma, mantle-cell lymphoma, and neuroblastoma cell lines [31]. Preliminary studies show that TIR-199 is active *in vivo* and arrests tumor cell growth in mice [30,31]. The current work builds on the initial studies with TIR-199 and shows that TIR-199 is indeed a dual constitutive and immunoproteasome inhibitor with high inhibitory activity against PSMB5 and PSMB8 subunits and can bypass bortezomib-resistant mutant of PSMB5. TIR-199 can induce cell death in myeloma, TNBC, and non-small cell lung cancer, and can trigger synergistic cytotoxicity in combination with DYRK2 inhibitor LDN192960. Although a highly hydrophobic natural product derivative, TIR-199 is specific and does not inhibit 50 kinases tested *in vitro* and effectively impedes myeloma-mediated bone degeneration *in vivo* at low doses. Overall, we report a highly specific, bioactive syrbactin-based proteasome inhibitor with potent anticancer properties.

## Materials and methods

### Materials

Antibody against 20S proteasome (#BML-PW8195) and purified human 20S proteasome (#BML-PW8720) were from Enzo Lifesciences. Flag-M2 antibody (#F3165) was from Merck Millipore. GAPDH antibody (#CB1001500UG) was from Calbiochem. Antibodies against p62 (#5114), p21 (#2947), ubiquitin (#3936), and IκBα (#9242) were from Cell Signaling.

### General methods

All recombinant DNA procedures, electrophoresis, Coomassie staining, immunoblotting, and FLAG affinity purification were performed using standard protocols. DNA constructs used for transfection were purified from *Escherichia coli* DH5α using Macherey-Nagel NucleoBond® Xtra Maxi kits according to the manufacturer’s protocol. PSMB5 A49T+A50V (A108T+A109V in full-length PSMB5) mutagenesis was performed using the QuikChange® site-directed mutagenesis method (Stratagene) with Q5 polymerase (New England Biolabs). All DNA constructs were verified by DNA sequencing. For qRT-PCR analysis, total RNA from HEK293 cells were isolated using the NucleoSpin RNA kit (Macherey-Nagel, Bethlehem, PA). cDNA was synthesized using the iScript kit (Bio-Rad). qRT-PCR analysis was performed using the SYBR® Premix ExTaq™ II (Takara) on Applied Biosystems 7500 Real-Time PCR System. Data were normalized to corresponding GAPDH levels. Primers used for hGAPDH (Forward: ACATCGCTCAGACACCATG; Reverse: TGTAGTTGAGGTCAATGAAGGG), hPSMB8 (Forward: CGGGTAGTGGGAACACTTATG; Reverse: TTGACAACGCCTCCAGAATAG), and hPSMB9 (Forward: GCTTCACCACAGACGCTATT; Reverse: GCAGTTCATTGCCCAAGATG) were purchased from IDT.

### Cell culture

All mammalian cells were grown in a humidified incubator with 5% CO_2_ at 37°C. HEK293, Hs578T, MDA-MB-231, SW1573, A549 cells were cultured in Dulbecco’s modified Eagle’s medium (DMEM, Gibco) supplemented with 10% FBS, 1% l-glutamine, and 1% penicillin and streptomycin. MM.1S, ANBL6, U266B1, RPMI-8226, 5TGM1-GFP, AHH1, MPC11, H460, H522, H1581 cells were grown in RPMI 1640 (Gibco) supplemented with 10% FBS, and 1% penicillin and streptomycin. MCF10A and 184B5 cells were cultured in DMEM/F-12 medium supplemented with 5% horse serum, 20 ng/ml EGF, 0.5 μg/ml hydrocortisone, 100 ng/ml cholera toxin, 10 μg/ml insulin, and 1% penicillin and streptomycin. Transient transfection of HEK293 with mammalian expression vectors were carried out using FuGENE transfection reagent according to the manufacturer’s protocol. Cell lines were purchased from ATCC, except 5TGM1-GFP (kind gift from Dr. Babatunde Oyajobi, University of Texas San Antonio, U.S.A.), MCF10A (kind gift from Dr. Alexandra Newton, University of California San Diego, (UCSD) U.S.A.), and ANBL6 (kind gift from Dr. Robert Orlowski, MD Anderson Centre, U.S.A.).

### Cytotoxicity assays

A total of 5,000–11,000 cells were plated per well in a 96-well plate. Four to six hours post-plating, TIR-199 was added to the cells in biological triplicates at various concentrations ranging between 0 and 500 nM final concentration with 0 nM being DMSO-treated control. Cell viability was then determined using CellTiter 96® AQueous Non-Radioactive Cell Proliferation Assay kit after 48 h of drug treatment. Absorbance was measured in a Tecan multi-well plate reader and data represented as relative to DMSO control.

### CD138^+^ myeloma cell purification

CD138^+^ myeloma cells were purified from the bone marrow aspirates which were obtained from HIPAA compliant de-identified consenting patients in accordance with Institutional Review Board protocol: 181027 approved on 9 October 2019 at UCSD. The bone marrow aspirates were kindly provided by Dr. Caitlin Costello, Moores Cancer Center, UCSD. CD138^+^ primary myeloma cells were purified from fresh bone marrow aspirates of multiple myeloma patients using EasySep™ Human Whole Blood and Bone Marrow CD138 Positive Selection Kit II (StemCell Technologies) following manufacturer’s instructions. Viable cells were collected for further analyses.

### Proteasome and immunoproteasome assays

For 26S constitutive proteasome activity assays, cells were lysed in 50 mM Tris/HCl (pH 7.5), 0.1% Nonidet P-40, 1 mM ATP, 10 mM MgCl_2_, 1:1000 β-mercaptoethanol, and a phosphatase inhibitor cocktail (10 mM NaF, 20 mM β-glycerophosphate). Proteasome peptidase activities from either 0.3–0.5 mg of cell lysates or FLAG-pulldown from 2 mg of cell lysates were assayed using 100 μM fluorogenic peptide substrate Suc-LLVY-AMC (Enzo Life Sciences) for 30 min. The measured activity was normalized against total protein concentration. Fluorescence signal in whole cells or cell lysates were quantified using a Tecan Infinite® M200 Pro multi-well plate reader.

Immunoproteasome activity assays were carried out as stated previously [32]. Briefly, HEK293 cells transiently overexpressing FLAG-tagged PSMB9 or PSMB8 were treated with IFNγ (100 ng/ml; 48 h) to induce expression of immunoproteasome. Cells were lysed in 50 mM Tris/HCl (pH 7.5), 0.1% Nonidet P-40, 1 mM ATP, 10 mM MgCl_2_, 1:1000 β-mercaptoethanol and a phosphatase inhibitor cocktail (10 mM NaF, 20 mM β-glycerophosphate). Immunoproteasomes were affinity purified using FLAG agarose from 2 mg of cell lysate. Peptidase activities of purified immunoproteasomes were assayed using 100 μM fluorogenic peptide substrates Ac-ANW-AMC for PSMB8 and Ac-PAL-AMC for PSMB9 (Boston Biochem) for 30 min. The measured activity was normalized against total protein concentration. Fluorescence signal in whole cells or cell lysates were quantified using a Tecan Infinite® M200 Pro multi-well plate reader.

### Animal study

NSG mice (NOD.Cg-Prkdcscid Il2rgtm1Wjl/SzJ; Stock: 005557) were purchased from the Jackson Laboratory. Mice were housed and maintained at the UCSD in full compliance with policies of the Institutional Animal Core and Use Committee (IACUC) protocol S03039 approved 31 March 2020. A total of 5 × 10^6^ MM.1S cells resuspended in sterile PBS were injected into the tail-vein of 8–12 weeks old *n*=6 NSG mice of either sex. After 7 days, the mice were randomized into two groups, *n*=3 each. TIR-199 was dissolved in 100% DMSO initially and further diluted down with 10 mM citrate buffer (pH 3.0) containing 10% (w/v) sulfobutylether-β-cyclodextrin (Carbosynth, U.S.A.) to a final DMSO concentration of 1%. Mice were injected at a daily intraperitoneal dose of 5 mg/kg or vehicle alone for 4 consecutive days. Seven days after dose completion, mice were killed initially under carbon dioxide followed by cervical dislocation. Both femurs were extracted rapidly and fixed in 10% formalin. Anesthesia was not required for the tail vein and intraperitoneal injections.

### Micro-computed tomography imaging and data analysis

Micro-computed tomography (μCT) imaging and data analysis was performed as stated previously [20] by investigators who were blinded to allocation. Femurs from MM.1S/NSG xenograft mice were fixed in 10% formalin for 24 h and washed 2× in PBS. The femurs were imaged with a μCT scanner, Skyscan 1076 (Kontich, Belgium). Each sample was wrapped in paper tissue that was moistened with PBS, and scanned at 9-μm voxel size, applying an electrical potential of 50 kVp and current of 200 μA, using a 0.5-mm aluminum filter. A beam hardening correction algorithm was applied prior to image reconstruction. Image data were visualized for each sample with Dataviewer and CTAn (Kontich, Belgium). A series of 2-D transaxial slices were generated over various regions of the femur. Fifteen slices were distributed around the center slice position with a separation of 0.06 mm for the metaphysis and mid-diaphysis regions, and 0.03 mm for the proximal femur region. Trabecular bone analysis was performed as appropriate for skeletally mature animals [33]. The trabecular region was selected by automatic contouring as close as possible to the periosteum but without overlapping the cortical bone. An adaptive threshold (using the mean maximum and minimum pixel intensity values of the surrounding ten pixels) was used to identify trabecular bone. From this region of trabecular bone, the tissue volume [24], trabecular bone volume (BV), trabecular bone volume fraction (% BV/TV), trabecular separation (Tb.Sp) and trabecular number (Tb.N) were determined. The technical quality of the image cross-sections was checked for damage before quantitative analysis was performed. Damaged areas were strictly avoided and never included in any quantitative analyses. Outliers were verified for legitimacy by checking the scan and reconstruction log file, image rotation, selection of the tip of growth plate, number of slices in metaphysis and diaphysis, and the contours.

### TIR-199 specificity profiling

TIR-199 specificity profiling assays were carried out at The International Centre for Protein Kinase Profiling (http://www.kinase-screen.mrc.ac.uk/). TIR-199 kinase specificity was determined against a panel of 50 protein kinases as described previously [34,35]. The assay mixes and ^33^P-γATP were added by Multidrop 384 (Thermo). Results are presented as a percentage of kinase activity in DMSO control reactions. Protein kinases were assayed *in vitro* with 1 μM final concentration of TIR-199 and the results are presented as an average of triplicate reactions ± SD or in the form of comparative histograms.

### *In silico* docking studies

Molecular docking simulation was performed using AutoDock Vina software [36]. AutoDock Tools (ADT) version 1.5.6 was used for structure pre-processing, while Discovery Studio Visualizer and PyMOL were used to analyze bound conformations and interactions as reported previously [37]. 3D structure of TIR-199 was drawn using ChemBio3D Ultra 11. To minimize energy, RMS gradient was set to 0.100 in each iteration. All structures were saved as SYBYL2 (.mol2) file format for input to ADT. Crystal structure of human immunoproteasome with all 14 subunits (Protein Data Bank (PDB) ID: 7AWE) was imported (as .pdb file) from the RCSB PDB (http://www.rcsb.org/). With the help of ADT, we extracted the crystal structure of PSMB8, PSMB9, and PSMB10 separately. Bound ligand and water molecules were removed, and the polar hydrogens were added. Finally, Gasteiger charges were added to each atom and the non-polar hydrogen atoms were merged to the protein structure. The structure was saved in PDBQT file format.

### Statistics and data presentation

Details of all statistical tests and multiple comparisons used to derive *P*-value has been detailed in figure legends. All experiments were repeated two to three times with multiple technical replicates to be eligible for the indicated statistical analyses, and representative image has been shown. All results are presented as mean ± SD unless otherwise mentioned. For animal studies, tumor-bearing mice of either sex were randomized into two equal groups of *n*=3 prior to vehicle or TIR-199 treatment. The investigators were blinded to allocation during μCT analysis and outcome assessment. Data were analyzed using GraphPad Prism statistical package.

## Results

### TIR-199 inhibits the constitutive 26S proteasome activity

**Figure 1:**
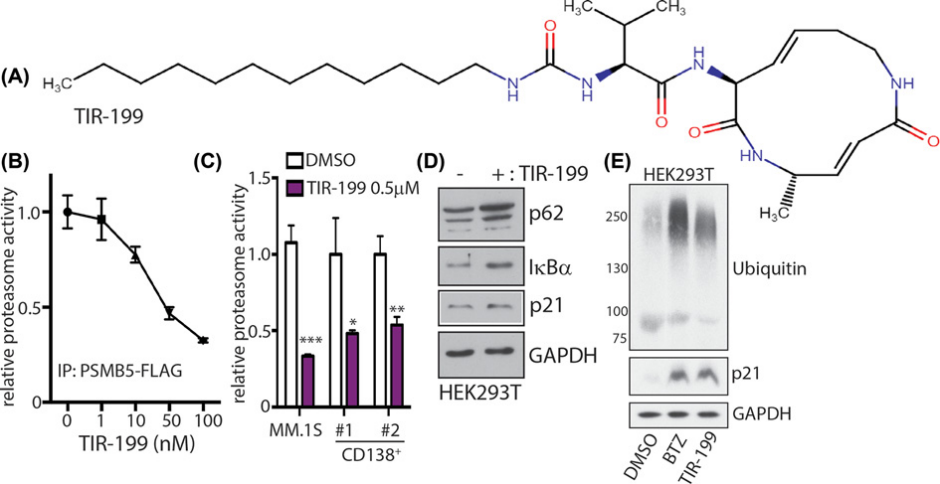
TIR-199 is a syrbactin-based constitutive proteasome inhibitor (**A**) Chemical structure of syrbactin derivative TIR-199. (**B**) Wildtype (WT) PSMB5-FLAG was immunoprecipitated from HEK293 cells stably expressing PSMB5-FLAG. PSMB5-FLAG was assayed using 100 μM Suc-LLVY-AMC in proteasome assay buffer with the indicated concentrations of TIR-199. Fluorescence signal upon release of AMC was measured in a Tecan plate reader. The results are presented as proteasome activity relative to the DMSO-treated control. Results are means ± S.D. for *n*=3 replicates. (**C**) Proteasome activity in lysates from MM.1S and primary CD138^+^ myeloma cells purified from two patients treated with DMSO or 0.5 μM TIR-199 for 0.5 h was measured with Suc-LLVY-AMC. *P<0.05; **P<0.01; ***P<0.001 (compared with control treated, two-tailed paired Student’s *t* test, mean ± SD from *n*=3 replicates). Also refer to Supplementary Figure S1. (**D)** HEK-293T cells were treated in the absence (DMSO) or presence of 0.5 μM of TIR-199 for 6 h. Cell lysates were subjected to immunoblotting with the indicated antibodies. Similar results were obtained in three independent replicates. (**E**) HEK-293T cells were treated in the absence (DMSO) or presence of either 0.1 μM of bortezomib (BTZ) or 0.2 μM of TIR-199 for 4 h. Cell lysates were subjected to immunoblotting with the indicated antibodies. Similar results were obtained in two independent replicates.

TIR-199 (Figure 1A) is a syrbactin analog which has previously been reported as a proteasome inhibitor [30]. TIR-199 potently inhibited the chymotryptic-like activity of immunoprecipitated PSMB5-FLAG with an IC_50_ of 50 nM (Figure 1B). Furthermore, consistent with previous studies [30], we observed inhibition of chymotryptic-like endogenous proteasome activity in TIR-199-treated myeloma MM.1S cell lysates (Figure 1C). To further investigate the ability of TIR-199 to inhibit proteasome activity in primary patient-derived myeloma cells, we obtained bone marrow samples from two refractory multiple myeloma patients with treatment history and recorded disease relapse. We purified the CD138^+^ myeloma cells from the bone marrow and then treated these cells with either DMSO-control or TIR-199 at 500 nM for 0.5 h, and then measured the chymotryptic-like proteasome activity. Upon treatment with TIR-199, the proteasome activity decreased significantly compared with DMSO (Figure 1C), suggesting that TIR-199 is effective at decreasing proteasome activity in myeloma patients with treatment history. To determine the efficacy of TIR-199-mediated inhibition of the endogenous proteasome, we treated HEK293T cells with 250 nM of TIR-199 for 6 h and conducted immunoblots to assess if proteasome substrates would accumulate over time. Indeed, proteasome substrates p62, IKBα, and p21 protein levels increased suggesting that the proteasome is inhibited by TIR-199 (Figure 1D). Like 100 nM bortezomib, HEK293T cells treated with 200 nM TIR-199 for 4 h led to the accumulation of ubiquitylated proteins (Figure 1E). Furthermore, TIR-199 is specific and does not inhibit 50 other kinases tested *in vitro* even at 20-fold higher concentration to PSMB5 IC_50_ (Supplementary Figure S1).

### TIR-199 inhibits the immunoproteasome

**Figure 2:**
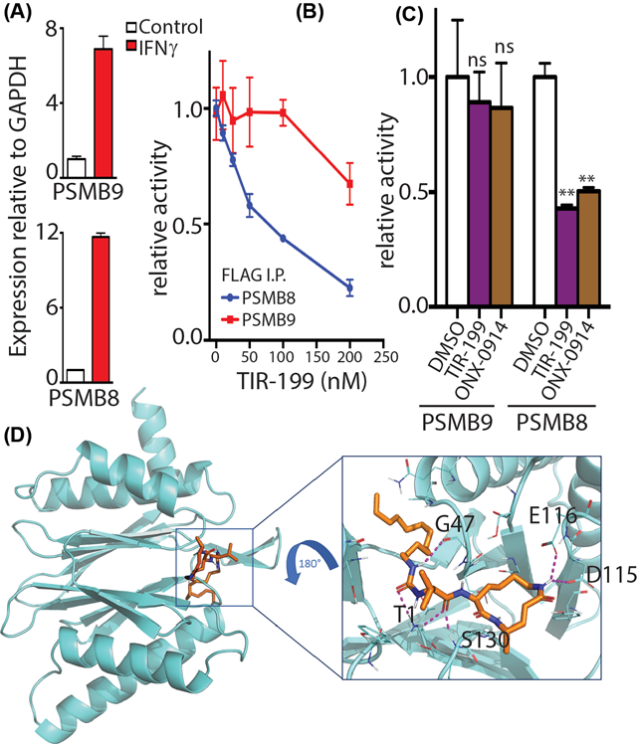
TIR-199 inhibits immunoproteasome subunit PSMB8 but not PSMB9 (**A**) Relative expression of PSMB8 and PSMB9 in HEK293 cells upon 100 ng/ml IFNγ stimulation for 48 h. (**B**) PSMB8 or PSMB9 was immunoprecipitated for 48 h in 100 ng/ml IFNγ-stimulated HEK293 cells transiently overexpressing either PSMB8-FLAG or PSMB9-FLAG. Activity of PSMB8-FLAG or PSMB9-FLAG were assayed using 100 μM of either Ac-PAL-AMC for PSMB9 or Ac-ANW-AMC for PSMB8 in proteasome assay buffer with the indicated concentrations of TIR-199. Fluorescence signal upon release of AMC was measured in a Tecan plate reader. The results are presented as proteasome activity relative to the DMSO-treated control. Results are means ± S.D. for *n*=3 replicates. (**C**) The FLAG immunoprecipitates as in (B) were treated with either DMSO or 100 nM TIR-199 or 100 nM ONX-0914 for 30 min and activity assays were carried out with respective fluorogenic peptide substrates. **P<0.01; ns, not significant (compared with DMSO control treated, two-way ANOVA with Tukey’s multiple comparisons, mean ± SD from *n*=3 independent replicates). (**D**) TIR-199 (orange/blue) was docked into the crystal structure of human PSMB8 (cyan). Identified amino acid interacting residues are highlighted in black. Hydrogen bonds between TIR-199 and the amino acids in the binding pocket are shown by red dashed lines. Also refer to Supplementary Figure S2.

HEK293 cells ectopically expressing PSMB8 or PSMB9 were treated with IFNγ for 48 h. A marked upregulation of PSMB8 and PSMB9 mRNA were observed which suggested immunoproteasome induction (Figure 2A). Flag agarose was utilized to immunoprecipitate PSMB8 or PSMB9 to pull-down the immunoproteasome. To determine how TIR-199 affects the immunoproteasome, an activity assay was conducted on PSMB8 using Ac-ANW-AMC as the substrate peptide and on PSMB9 using Ac-PAL-AMC. TIR-199 potently inhibited the chymotryptic-like activity of immunoprecipitated PSMB8-FLAG with an IC_50_ of ∼100 nM while caspase-like activity of PSMB9 seemed largely more resistant to TIR-199 even at 250 nM (Figure 2B). Next, we compared the immunoproteasome inhibitory activity of TIR-199 with specific PSMB8 inhibitor ONX-0914 [12]. Indeed, 100 nM TIR-199 addition inhibited PSMB8 potently and similarly to ONX-0914. However, like ONX-0914, TIR-199 at 100 nM did not inhibit PSMB9 activity suggesting a PSMB8-specific effect for TIR-199 (Figure 2C). To further predict TIR-199 binding to the immunoproteasome, *in silico* binding studies were conducted (Figure 2D). Docking analysis showed that TIR-199 formed six hydrogen bonds with Gly^47^, Thr^1^, Ser^130^, Glu^116^, Asp^115^ of PSMB8 with Thr^1^ forming two hydrogen bonds with the same bond length of 2.3 Å (Supplementary Figure S2A). Furthermore, ten alkyl interactions through the dodecyl group of TIR-199 and multiple Van der Waals interactions enhanced the stability of the predicted TIR-199-bound PSMB8 structure (Supplementary Figure S2A). Interestingly, the predicted structure of TIR-199 and PSMB9 exhibited only three hydrogen bonds (Supplementary Figure S2B) while TIR-199 and PSMB10 exhibited four hydrogen bonds (Supplementary Figure S2C).

### TIR-199 causes cytotoxicity in cancer cells

**Figure 3:**
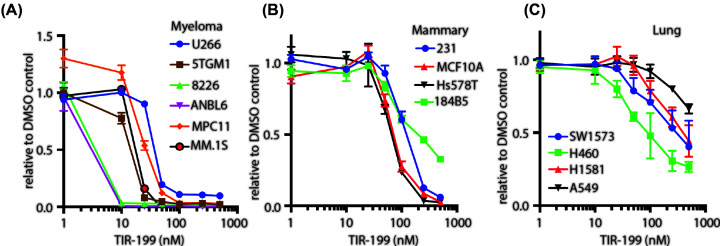
TIR-199 causes cell death TIR-199 induces cytotoxicity in various (**A**) multiple myeloma, (**B**) TNBC, and (**C**) lung cancer cell lines after 48-h incubation. MTS absorbance was measured in a Tecan plate reader. Cell viability was ascertained with CellTiter 96 AQueous Non-Radioactive Cell Proliferation Assay kit. Data were represented as percent viable compared with DMSO-treated cells. Results are means ± S.D. for *n*=3 replicates.

It has been shown previously that TIR-199 can cause cytotoxicity in multiple myeloma cell lines MM1.S and U266 [31]. We conducted cytotoxicity assays on a panel of human and murine multiple myeloma cell lines (Figure 3A). Similar to most proteasome inhibitors reported till date, TIR-199 killed myeloma cells with an EC_50_ ranging between 1 and 50 nM. Similar to myeloma, TNBC cells have also been reported to be particularly sensitive to proteasome inhibition [22]. Therefore, we expanded the study to include TNBC cells and report the EC_50_ values for TIR-199 to be between 50 and 300 nM (Figure 3B). Although lung cancer cell lines have not been reported to be sensitive to proteasome inhibition, a case study did report exceptional therapeutic benefits of bortezomib in a KRAS-mutated lung adenocarcinoma patient [24]. Although within the lung cancer panel, EC_50_ values ranged from 100 to >1000 nM for TIR-199, H460 was found to be the most sensitive lung cancer cell line to TIR-199 inhibition at 100 nM EC_50_ (Figure 3C).

### TIR-199 bypasses PSMB5 mutation-driven bortezomib resistance and induces cytotoxicity in combination with DYRK2 inhibitor

**Figure 4:**
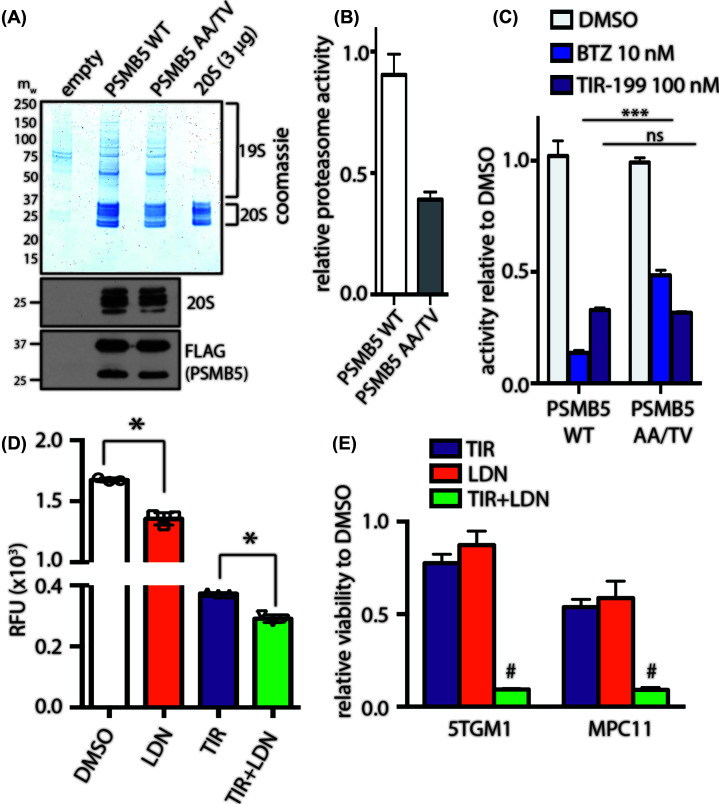
TIR-199 bypasses bortezomib-resistant PSMB5 AA/TV mutation and induces synergistic cytotoxicity in myeloma cells with LDN192960 (**A**) WT and AA/TV mutated PSMB5-FLAG were immunoprecipitated from HEK-293 cells stabling expressing the wildtype and mutant enzymes and were analyzed by Coomassie Blue staining of a polyacrylamide gel (top panel) and immunoblotting with indicated antibodies (bottom panel). Purified 20S proteasome was loaded as control to predict amount. (**B**) Intrinsic proteasome activities of the equivalent amounts of PSMB5 and PSMB5 (AA/TV) were compared by carrying out a proteasome activity assay using Suc-LLVY-AMC substrate peptide. Values are means ± S.D. for an experiment carried out in triplicate. (**C**) Proteasome activities of the equivalent amounts of PSMB5 and PSMB5 (AA/TV) were compared in the presence or absence of indicated molecules by carrying out a proteasome activity assay using Suc-LLVY-AMC substrate peptide. Data represented as activity relative to respective DMSO-treated sample. ***P<0.001; ns, not significant (compared with DMSO control treated, two-way ANOVA with Tukey’s multiple comparisons, mean ± SD from *n*=3 independent replicates). (**D**) MM.1S cells were pretreated with indicated drugs or combination (TIR, TIR-199; LDN, LDN192960) for 1 h, and proteasome activity was measured in cell lysates using Suc-LLVY-AMC. *P<0.05 (compared with control-treated, ordinary one-way ANOVA, mean ± SD from *n*=3 independent experiments). (**E**) Multiple myeloma cells MPC11 and 5TGM1 were treated with TIR-199 alone (10 nM for 5TGM1 and 25 nM for MPC11) or with LDN192960 alone (3 μM for 5TGM1 and 5 μM for MPC11) or the combination of TIR-199 and LDN192960 for 48 h, and cell viability was analyzed by CellTiter 96 AQueous Non-Radioactive Cell Proliferation Assay kit. Viability of DMSO-treated cells was utilized as control. Data are represented as fold viability of DMSO-treated control for each cell line (# indicates statistical significance compared with each single drug treatment; two-way ANOVA with Sidak’s multiple comparison).

A recurrent bortezomib-resistant PSMB5 mutation observed in cells upon prolonged bortezomib treatment is the dual amino acid substitutions A49T and A50V (AA/TV), which directly affect bortezomib binding [38] and also reduces specific activity of the proteasome [8]. Since TIR-199 has an altered binding mechanism to PSMB5 compared with bortezomib and does not exhibit direct hydrogen bond interaction with Ala^49^ [27], we hypothesized that AA/TV harboring cells will exhibit similar sensitivity to TIR-199 as parental cells. We generated HEK293 cells with stable ectopic overexpression of wildtype (WT) or AA/TV PSMB5 with a C-terminal FLAG tag. We utilized FLAG tag pull-down to immunoprecipitate the 26S proteasome and observed similar proteasomal subunit distribution on a Coomassie-stained gel for both WT and AA/TV (Figure 4A). When we further assayed proteasome activity on the immunoprecipitates using the peptide substrate Suc-LLVY-AMC, we observe reduced proteasome activity in the AA/TV mutant compared with WT (Figure 4B) which is consistent with previous report. In order to determine sensitivity of AA/TV PSMB5 toward TIR-199, the immunoprecipitated PSMB5 was treated with either TIR-199 or bortezomib. Treatment of WT PSMB5 with 10 nM bortezomib led to a dramatic reduction in proteasomal activity, while AA/TV mutant exhibited significant resistance (Figure 4C). However, both WT and AA/TV harboring cells were equally sensitive to 100 nM TIR-199 (Figure 4C). This suggests that TIR-199 binds to the constitutive proteasome via a different mechanism than bortezomib, allowing it to bypass bortezomib resistance. Interestingly, a combination of TIR-199 with DYRK2-inhibitor LDN192960 exhibited a modest additive effect toward the biochemical inhibition of proteasome activity in MM.1S cells (Figure 4D). Next, we wanted to determine if LDN192960-TIR-199-mediated additive impairment of proteasome activity could have an effect on cancer cell viability. Interestingly, a remarkable synergistic cytotoxicity was induced in proteasome-addicted MM cell lines MPC11 and 5TGM1 upon treatment with LDN192960 and TIR-199 (Figure 4E).

### TIR-199 impedes multiple myeloma progression *in vivo*

**Figure 5:**
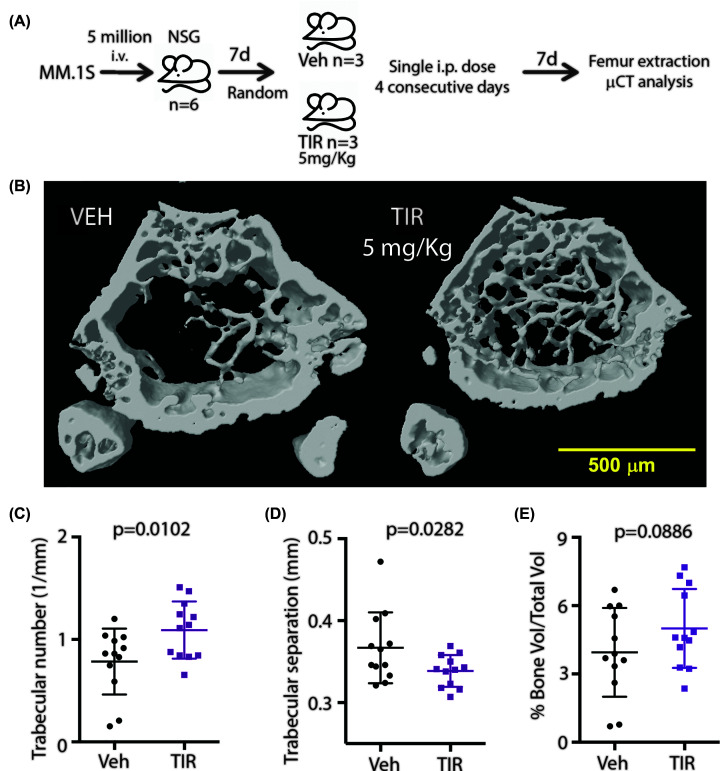
Low-dose TIR-199 delays myeloma-mediated bone degeneration (**A**) MM.1S cells were injected i.v. into NSG mice *n*=6. One-week post-injection, mice were randomized into two groups of *n*=3 and treated with vehicle or TIR-199 5 mg/kg i.p. daily for four consecutive days. One-week post-treatment, the mice were killed, and formalin-fixed femur bones were imaged using μCT. (**B**) Representative μCT image is shown post-μCT the trabecular number (**C**), the trabecular separation (**D**), and percentage of bone volume over total volume (**E**) were quantified for distal and proximal femurs.* P*-value shown for vehicle versus TIR-199 treatment and derived using *t* test. Data represented as mean ± SD, from *n*=6 femurs combining distal and proximal regions’ analyses for each.

Previous studies have shown that TIR-199 is active *in vivo* and can reduce cancer cell proliferation in hollow fiber assays [30] and an ectopic tumor xenograft model [31]. Although TIR-199 can reduce ectopic tumors, no information is available regarding the ability of TIR-199 in targeting systemic myeloma-mediated bone degeneration *in vivo* which is the major hallmark of multiple myeloma. To address this, we intravenously injected MM.1S cells into NSG mice of either sex to generate a systemic multiple myeloma model exhibiting the canonical myeloma-induced systemic bone degeneration. Mice were randomized 1 week post-intravenous MM.1S implantation and *n*=3 mice were treated with either vehicle control or TIR-199 at 5 mg/kg per day for four consecutive days. We used a lower dose keeping in mind the relatively toxic effects of systemic proteasome inhibition. Seven days post treatment-completion the femurs were collected, fixed, and μCT quantitative analysis was performed on the femur pairs of three vehicle-treated and three TIR-199-treated mice (Figure 5A). Trabecular bone was quantified in the distal metaphysis and proximal regions independently and data were presented by combining both analysis for each cohort to provide a more holistic view. Representative μCT image of femur cross-sections are shown (Figure 5B). Quantitative analysis of the trabecular bone in both the distal and proximal regions of the femurs presented a higher trabecular number (Figure 5C) which is the number of traversals across a trabecular region per unit length on a random linear path through the volume of interest. The TIR-treated mouse femurs also exhibited significantly lower trabecular separation (Figure 5D) which is the thickness of spaces between trabecular bone. Although the combined distal and proximal numbers for % bone volume over total volume was not significant (Figure 5E), the TIR-199-treated mouse femurs had less damage. This suggests that TIR-199 treatment at a low dose of 5 mg/kg for 4 days had slowed down the myeloma-mediated bone degeneration process.

## Discussion

In the current study we extensively characterize syrbactin-class molecule TIR-199 as a potent constitutive proteasome (Figure 1) and PSMB8 immunoproteasome subunit inhibitor (Figure 2). We further establish that TIR-199 treatment potently reduced proteasome activity in patient-derived primary myeloma cells, (Figure 1C) and leads to accumulation of proteasome substrates in cells (Figure 1D,E). Previous co-crystallography studies showed that syrbactin-based molecules use a covalent interaction with PSMB5 Thr^1^ residue to stabilize the inhibitor bound structure [27]. Our *in silico* docking studies with immunoproteasome subunits predicted that Thr^1^ on PSMB8 is also involved in binding to TIR-199 (Figure 2D). However, bortezomib binding involves a critical hydrogen bond with Ala^49^ on PSMB5 [38] which is not observed in case of TIR-199 (Figure 2D and Supplementary Figure S2A) which explains the mechanism by which TIR-199 is able to bypass bortezomib resistance in the AA/TV PSMB5 mutation (Figure 4C). Indeed, a previous study has reported bortezomib-resistant myeloma and mantle-cell lymphoma cells were sensitive to TIR-199 [31]. Furthermore, TIR-199 clearly prefers the PSMB8 subunit over PSMB9 (Figure 2B) and PSMB10 and this is probably due to the reduced number of hydrogen bonds predicted for TIR-199-PSMB9 or PSMB10 interactions (Supplementary Figure S2B,2D). Inhibition of dual classes of proteasomes by TIR-199 could bode well for patients since epoxyketone-class dual inhibitor oprozomib showed initial promise in clinical trials [11]. TIR-199 could expand the repertoire of proteasome-based therapeutics since further drug resistance is expected to appear for the next generation of boronates and epoxyketones as well. The ability of TIR-199 to induce cytotoxicity in multiple myeloma, TNBC, and lung cancer lines (Figure 3) further attests to its potency and the fact that the molecule can synergistically induce myeloma cell death in combination with DYRK2 inhibitor LDN192960 (Figure 4D,E) further opens new possibilities in therapeutic targeting of proteasome regulators. Proteasome addiction is well documented for myeloma and TNBC cells, while more recently, a small proof-of-concept human trial showed that some lung adenocarcinoma cancer patients with the KRAS G12D mutation responded well to proteasome inhibitor therapy [24]. In our study, we found that lung cancer cell line H460, which harbors a KRAS Q61H mutation, was maximally sensitive to TIR-199 within the lung cancer panel (Figure 3C). Paradoxically, KRAS G12C harboring A549 cells seemed relatively more resistant (Figure 3C). This suggests that proteasome targeting may have a broader therapeutic potential than previously appreciated and further work is needed to establish a potential link between KRAS mutations and proteasome-inhibitor sensitivity.

Various kinases have been reported to regulate proteasome activity including DYRK2 [14], PIMs [17], PKA [15], and PKG [16]. In fact PIM kinases are currently being pursued as active targets for multiple myeloma [39] and it would be ideal to study the cytotoxic potency of TIR-199 in combination with similar proteasome-regulating kinase inhibitors to ascertain the ideal therapeutic regimen for the future. As a monotherapy option, TIR-199 has been shown to reduce ectopic tumor volume previously [31] while we show that dosing even at one-fifth the maximal tolerated dose, TIR-199 can significantly delay systemic myeloma-mediated bone degeneration in an orthotopic mouse xenograft model (Figure 5). Mice treated with TIR-199 showed improved trabecular bone numbers (Figure 5C) and smaller gaps between the bones (Figure 5B,D,E) suggesting improved bone health compared with vehicle-treated control and consequently reduced myeloma burden. Since we were limited by drug availability, we could not study the beneficial effect of TIR-199 treatment on overall survival over a longer period, however, our data clearly suggests that TIR-199 exhibits all the *bona fide* hallmarks of a novel class of proteasome inhibitor.

With over 160,000 patients worldwide, multiple myeloma accounts for nearly 10% of all hematological malignancies [40]. Patients exhibit extensive drug resistance with high therapy-induced toxicities [7,13]. Hence, establishing novel syrbactin class TIR-199 could indeed expand the therapeutic option for refractory patients and extend their survival considerably. TIR-199 is currently being developed by Lodo Therapeutics, U.S.A. to expand their preclinical pipeline with selective peptide-based proteasome inhibitors with potential application in solid tumors and other indications. TIR-199 is hydrophobic, has a high LogP value at 5.9, molecular weight of 533.7 g/mol, does not adhere to Lapinski’s rule of five and also presents a high topological polar surface area of 128 angstrom squared. Although highly active *in vivo*, the robust and complex structure might hinder pharmacokinetics and solubility in human trials and may require optimizing delivery vehicles for full bioactivity. Further work is needed to translate syrbactin-class proteasome inhibitors to the clinic, however, the lead compound TIR-199 is indeed a promising drug for the near future.

## Supplementary Material

Supplementary Figures S1-S2

## Data Availability

All data that support these findings of the present study are included in this manuscript. Further information and reagents are available upon request to the corresponding author, Sourav Banerjee. TIR-199 has been licensed to Lodo Therapeutics, U.S.A. and not available from the authors. TIR-199 structure has been deposited at PharmaCompass: https://www.pharmacompass.com/chemistry-chemical-name/tir-199.

## Open Access

Open access for this article was enabled by the participation of University of Dundee in an all-inclusive *Read & Publish* pilot with Portland Press and the Biochemical Society under a transformative agreement with JISC.

## Abbreviations

ADT: AutoDock Tools
DMEM: Dulbecco’s modified Eagle’s medium
PDB: Protein Data Bank
TNBC: triple-negative breast cancer
UCSD: University of California San Diego
μCT: micro-computed tomography
