# Supplementary material for: Syrbactin-class dual constitutive- and immuno-proteasome inhibitor TIR-199 impedes myeloma-mediated bone degeneration in vivo
Source: Biosci Rep. 2022 Feb 11;42(2):BSR20212721. doi: 10.1042/BSR20212721 (PMC8837819; doi:10.1042/BSR20212721)
Supplement: Supplementary Figures S1-S2 [file BSR-2021-2721_supp.pdf]

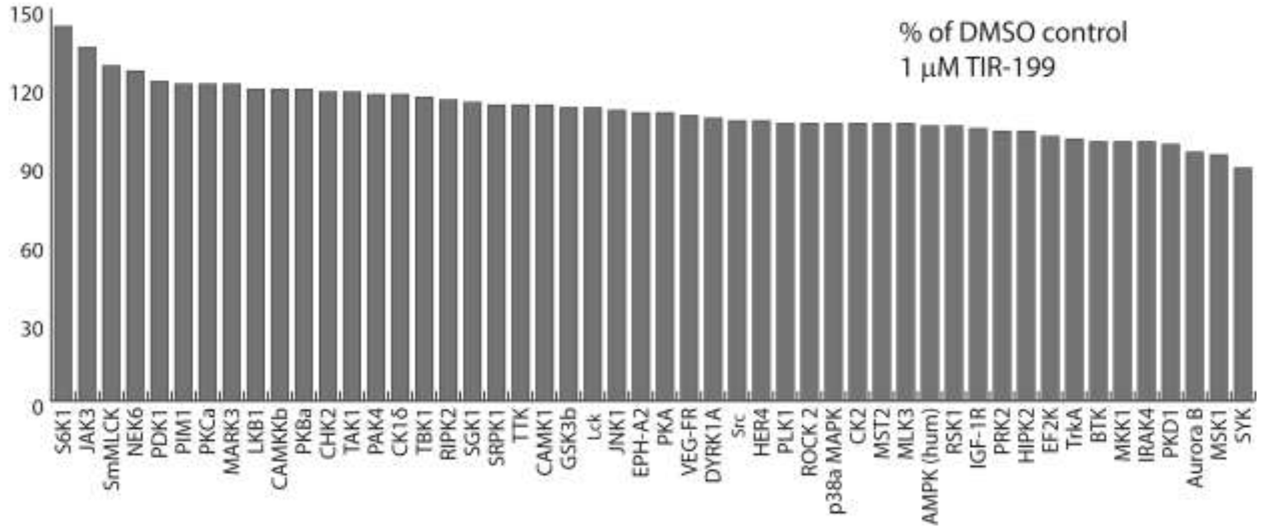

Supplementary Figure S1 TIR-199 does not inhibit the activity of 50 kinases in vitro. Kinase profiling of TIR-199 at 1 µM was carried out against the panel of 50 kinases at the International Centre for Protein Kinase Profiling (http://www.kinase-screen.mrc.ac.uk/).

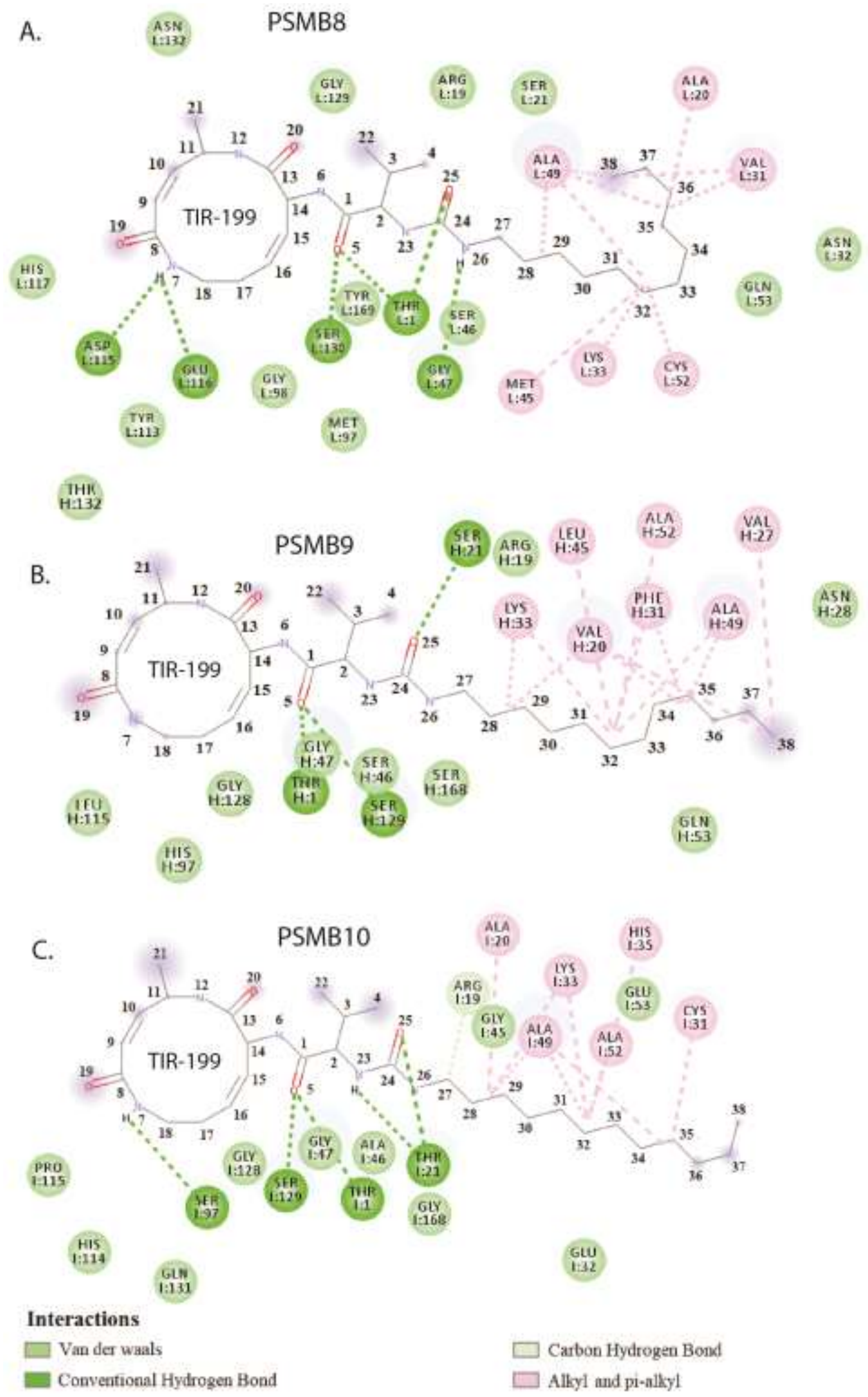

Supplementary Figure S2 TIR-199 is predicted to form more hydrogen bonds while binding to PSMB8 compared to PSMB9 or PSMB10. 2D interaction diagram showing TIR-199 docking mechanism in the active site of PSMB8 **(A),** PSMB9 **(B)**, PSMB10 **(C).**
